# Palliative care to cancer patients: how COVID-19 pandemic could affect quality of care

**DOI:** 10.31744/einstein_journal/2022AO6459

**Published:** 2022-05-18

**Authors:** Juliana Todaro, Camila Viale Nogueira, Elisa Rossi Conte, Rafael Aliosha Kaliks

**Affiliations:** 1 Hospital Israelita Albert Einstein São Paulo SP Brazil Hospital Israelita Albert Einstein, São Paulo, SP, Brazil.

**Keywords:** Palliative care, Neoplasms, Coronavirus infections, COVID-19, SARS-CoV-2, Betacoronavirus, Quality of health care

## Abstract

**Objective:**

To evaluate the impact of COVID-19 pandemic on the care delivered to hospitalized cancer patients in end-of-life.

**Methods:**

A retrospective analysis of data of hospitalized patients with advanced solid tumors, who died under exclusive palliative care during first wave (March 2020 to July 2020) compared with the period previous pandemic (January 2018 to February 2020).

**Results:**

A total of 190 oncologic patients were included, 161 patients before the pandemic, and 29 in the period from March 2020 to July 2020. The average hospitalization was 497.2 patients per month, before the pandemic, and dropped to an average of 46.5 in the pandemic, whereas the death rate decreased from an average of 6.3 patients per month to 4.8. Considering the benchmarks for quality of care during end-of-life care, preferences on life assistance were discussed prior to hospitalization for 34.4%, before the pandemic, and 13.8% during the pandemic (p=0.0298); 9.3% received chemotherapy 15 days prior to the date of death, before the pandemic, and 20.7%, in the pandemic (p=0.1012).

**Conclusion:**

Based on the present results, despite the decrease in oncology admissions, the advanced-stage cancer patients continued to seek hospital for end-of-life care. However, we could observe in our benchmarking analyses for palliative quality of care that talks about prognosis occurred less often during the pandemic.

## INTRODUCTION

On March 11, 2020, the World Health Organization (WHO) officially declared the pandemic caused by the new coronavirus disease 2019 (COVID-19). Since then, efforts have been made to prevent spread of the virus, especially among the vulnerable population, where disease-related morbidity and mortality is most pronounced.^( 1 - 3 )^

The vulnerable population consists of patients aged over 60 years, or those who suffer from chronic diseases and comorbidities. Among them, patients on treatment for cancer, whose immune response to pathogens may be highly impaired.^( 3 - 7 )^

Considering person-to-person transmission, oncology services have taken decisions based on expert opinions to avoid infection by COVID-19. Cancer centers expanded the use of telemedicine; postponed surgery and adjuvant therapies; delayed radiation therapy; switched to oral drugs whenever possible; expanded prophylaxis for neutropenia, and reviewed the duration of maintenance treatment.^( 8 , 9 )^

Despite the risks related to hospitalization, during oncology care patients still require admission due to treatment-related complications, or for specific care, such as the cases of end-of-life care.^( 10 , 11 )^An analysis already described 75% of patients with advanced stages of neoplasms required hospitalization in the last year of life.^( 10 )^ The demand for hospital services by these patients is also associated with the feeling of security.^( 11 )^

In COVID-19 pandemic, even in this group of patients, protective barriers were implemented with some actions, such as symptom surveillance, supportive measures to decrease toxicity of palliative chemotherapy, and encouraging home care. If on the one hand these barriers were done to protect patients and staff from COVID-19 transmission, on the other hand we did not know how this could affect the scenario of care for oncology patients with advanced disease.^( 8 , 9 )^

## OBJECTIVE

To evaluate the impact of COVID-19 pandemic on the care delivered to hospitalized cancer patients in end-of-life.

## METHODS

### Population recruiting and data collection

A retrospective analysis was conducted using data from hospitalized patients with advanced and incurable solid tumors, who died while on exclusive palliative care, in a ward dedicated to cancer treatment, in a tertiary hospital, in São Paulo, SP, Brazil, from March 2020 to July 2020. The period of choice represents the first 3 months of the COVID-19 pandemic. In this period, hospital made adjustments in routine to adapt the care to the outbreak.

Since the population of this study has the same characteristics of previous authorized research (CAAE: 32849820.8.0000.0071; protocol # 4.072.563), after the Ethics Committee approval for the proposed analysis of this study (CAAE: 14699219.4.0000.0071; protocol # 4.477.120), an analysis was conducted comparing the period before and after pandemic, here described: January 2018 to February 2020 and March 2020 to July 2020. Waiver of the Informed Consent was requested because both studies have as a requirement that the patient has died for their inclusion.

Patients’ data were obtained through medical records, described in open and structured fields. The variables defined were population and cancer diagnostic characteristics: age, sex, histological subtype of cancer, number of systemic treatment lines performed, subtype of the last treatment performed; date of hospitalization and date of death; reason for hospitalization; hospitalization secondary to cancer; cancer directed therapy within the last fifteen days before date of death, cancer directed therapy during hospitalization before death, nutritional care, time of palliative care team support, admission to intensive care unit (ICU); end-of-life care (discussion of life assistance preferences).

Information on time to define end-of-life care preferences was collected from unstructured fields, and categorized into 3 days before death, 7 days before death, and before last hospitalization.

The variables cancer-directed therapy within the last 15 days before date of death, time of palliative care team support, and discussion of preferences for end-of-life care address the recommendations of the oncology societies about what would be considered a supported practice and quality of care, in the course of neoplastic treatment and outcomes.^( 12 , 13 )^

### Statistical analysis

Descriptive analyses were made and qualitative data are described by absolute and relative frequencies, whereas quantitative data are described by means and standard deviations, or medians and quartiles, depending on the distribution observed in the sample. Minimum and maximum values observed in the sample were also presented.

For analysis, frequency results were compared using the Fisher’s exact test, and means using Student’s *t* test for two independent samples, or Wilcoxon’s non-parametric test, whenever appropriate. To demonstrate change in care over time, ARIMA Time Series Analysis model was performed. Significance level considered was 0.05, which is equivalent to a 95% confidence interval. The software JMP Pro version 13 (SAS Institute Inc., Cary, NC, United States, 1989-2019) was employed for this analysis.

## RESULTS

**Figure 1:**
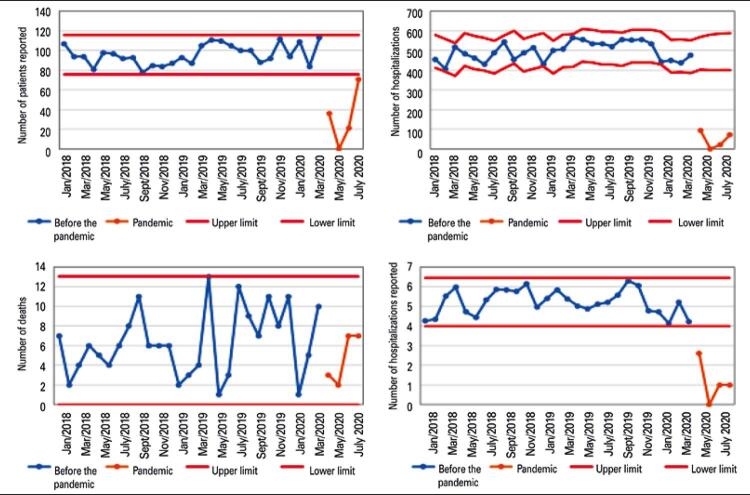
Time series and forecast limits for indicators

From March 2020 to July 2020, the number of admissions to the oncology ward dropped from a mean of 497.2 per month to 46.5 (9% of previous valor), and the number patients with advanced and incurable solid tumors who died while on exclusive palliative care in this unit dropped from a mean of 6.3 per month to 4.8 (76% of previous valor) ( Figure 1 ). The mean number of patients who were admitted to the oncology ward more than once a month was 5.2 before the pandemic, and 1.1 during this period (p=0.037).

**Figure 2:**
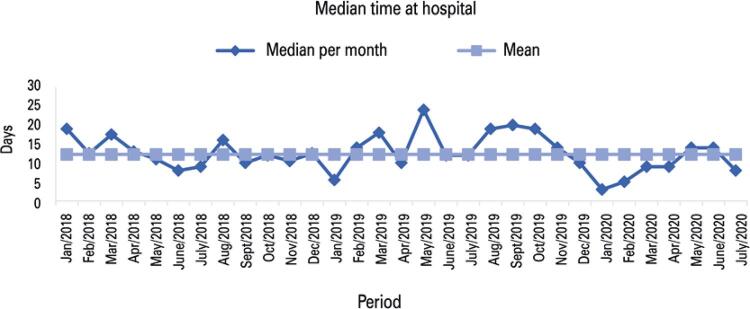
Median length of hospital stays

Before the pandemic, the median duration of the last hospitalization was 12.5 days per month in 2018, 14 days per month in 2019, and from January 2018 to July 2020, 9 days per month. From March 2020 to July 2020, the median was 9 days ( Figure 2 ).

A total of 190 oncology patients who died while on exclusive palliative care, between January 2018 and July 2020, were included. The reason for admission in 85% of them was symptom control. Of these, 161 patients were included before the COVID-19 pandemic, and 29 patients from March 2020 to July 2020. The sex distribution was 72 (44.7%) females and 89 (55.3%) males before the pandemic, and 17 (58.6%) females and 12 (41.4%) males during the pandemic (p=0.2250). The median age was 66.9 in the first period (mean of 66.9; standard deviation of 14.6; 22-95) and 64.6 in the second period (mean of 65; standard deviation of 15.5; 32-90).

**Figure 3:**
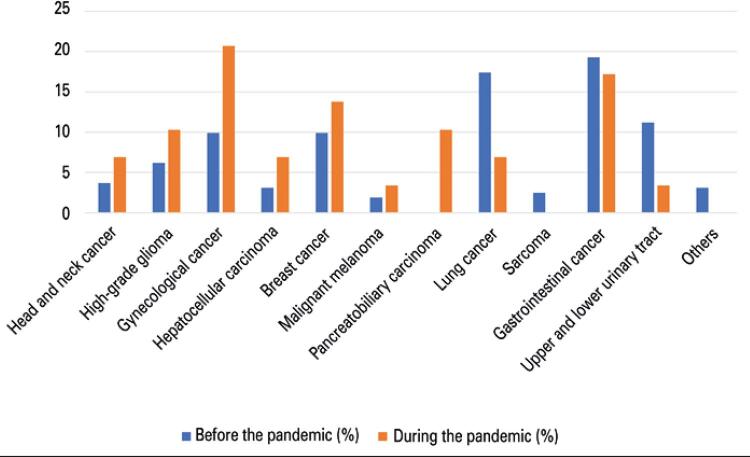
Distribution by subtype of neoplasm

The histological subtypes visualized in the period are similar, with 168 patients known to be metastatic. The average number of antineoplastic treatments in the period was 2.3 lines ( Figure 3 ). In the pandemic, only one patient did not have metastatic disease, and the treatment lines average among metastatic patients was 2.6.

**Figure 4:**
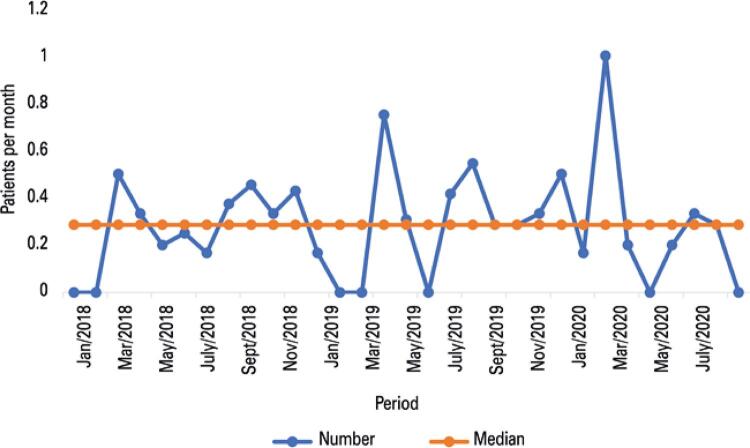
Number of patients referred to the intensive care unit

End-of-life care preferences were discussed before hospitalization for 55 patients (34.4%) before the pandemic, and 4 (13.8%) during the pandemic. Previously to the pandemic, 15 out of 161 (9.3%) patients received chemotherapy 15 days before death, and 13 of 161 (8.1%) patients received chemotherapy during the last hospitalization. During the pandemic, 6 out of 29 (20.7%) received chemotherapy 15 days before death, and 5 out of 29 (17.2%) patients received chemotherapy during the last hospitalization (p=0.1012). A total of 54 (33.5%) patients were referred to the ICU before the pandemic, and 5 (17.2%) in the pandemic (p= 0.0864) ( Figure 4 ). Eleven (6.8%) patients were given artificial nutrition support in the last thirty days of life before the pandemic, and two (6.9%) patients during the pandemic (p=1.0) ( Table 1 ).

**Table 1:** Characteristics before and during the pandemic

Characteristics	Before the pandemic	During the pandemic	Total	p value
Chemotherapy up to 30 days				
Yes	48 (30.0)	11 (37.9)	59 (31.2)	0.3926
No	112 (70.0)	18 (62.1)	130 (68.8)	
Chemotherapy up to 15 days				
Yes	15 (9.3)	6 (20.7)	21 (11.1)	0.1012
No	146 (90.7)	23 (79.3)	169 (88.9)	
Chemotherapy in last hospitalization				
Yes	13 (8.1)	5 (17.2)	18 (9.5)	0.1603
No	148 (91.9)	24 (82.8)	172 (90.5)	
Palliative care team support				
Yes	95 (59.0)	18 (62.1)	113 (59.5)	0.8388
No	66 (41.0)	11 (37.9)	77 (40.5)	
Referred to the intensive care unit				
Yes	54 (33.5)	5 (17.2)	59 (31.1)	0.0864
No	107 (66.5)	24 (82.8)	131 (68.9)	
Discussion about preferences up to 3 days before death				
Yes	136 (84.5)	25 (86.2)	161 (84.7)	1.0000
No	25 (15.5)	4 (13.8)	29 (15.3)	
Discussion about preferences before hospitalization				
Yes	55 (34.4)	4 (13.8)	59 (31.2)	0.0298
No	105 (65.6)	25 (86.2)	130 (68.8)	

Results expressed as n (%).

None of the patients hospitalized from March 2020 to July 2020 had been treated with radiation therapy in the last admission; before this period, the number was 4.4% (7 out of 152 patients; p=0.5978).

### Palliative care and support during the pandemic

A total of 113 (59.5%) of patients were supported by the palliative care team; in that, 95 before the pandemic, and 18 during this period, with no statistical difference, p=0.8388.

Comparing the group that received follow-up with the group that did not receive a visit from the palliative care team, in the period from January 2018 to July 2020, the group being followed by the team had their documentation regarding end-of-life preferences performed earlier than the group without. When the interval between the first palliative care visit and death is considered, the mean days of establish of preferences about end-of-life care assistance was 53.9 in the group with palliative care team *versus* 20.7 in the group with no such support (p=0.0223) ( Table 2 ).

**Table 2:** Documentation on end-of-life care preferences and palliative care

Measure	Mean±standard deviation	Median (Q1-Q3)	p value
Discussion about preferences up to 3 days before death			
No palliative care	33.9±80.5	1 (0-18.75)	0.5105
With palliative care	30.5±71.1	4 (0-32.75)	
Discussion about preferences up 7 days before death			
No palliative care	19.5±64.8	0 (0-11)	0.0036
With palliative care	37.7±75.9	9 (0-47.75)	
Discussion about preferences during the previous hospitalization			
No palliative care	20.7±51.6	2 (0-19)	0.0223
With palliative care	53.9±101.2	10 (0-63)	
Intensive care unit			
No palliative care	29.3±71.8	3.5 (0-27.25)	0.5929
With palliative care	34.9±73.9	5 (0-50.25)	

When evaluating the pandemic period with the prior period, this value is significant for the documentation prior to the hospitalization of the death. The number of patients whose preferences were recorded up to 7 days before death was 70,5% in the period previous pandemic *versus* 53.2% during (p=0.0208). No difference between admissions to the ICU was observed, among palliative support assistance in both periods.

During the pandemic, no significant difference between palliative pharmacological sedation for terminal ill patients was observed. The figure before the pandemic was 60.2%, and during the pandemic, 48.3% (p=0.3061).

## DISCUSSION

The COVID-19 pandemic has changed the health system, mainly in the first wave, when uncertainties were prevalent. For the first time, hospitals were an unsafe place to go, although many protective measures were promptly adopted: number of visitors was reduced, use of masks was required, hospitalization was avoided, and discharge planning was even more essential.^( 14 )^

Based on these, our study reported how this new scenario have affected the delivered of care to cancer patients in end-of-life in an oncology ward of a dedicated private hospital in São Paulo. To emphasize the period of adjustment, we choose to analyze data from March 2020 to July 2020. During this period, new routines were adopted to assist COVID-19 pandemic, but also, we needed to maintain non-COVID patients in safe. After July, our flows were better defined, and resume have started.

Thus, in our study, we observed that despite the feeling of insecurity related to the hospital environment, in the group of patients with advanced cancer and terminal disease, the decrease in the number of hospitalizations was not so pronounced. While the number of patients admitted to the oncology ward reduce to 9% of the previous valor, the number of patients who died there, in the chosen period, only dropped to 76% of the previous valor. These findings can be considered multifactorial, which includes the lack of culture in Brazil of dehospitalization to home care and hospices, especially at the end of life.

In addition, even before COVID-19, Henson et al. related this type of behavior among palliative patients to anxiety referring to the disease, previous pattern of seeking this service in crisis situations, feeling of security on the part of patients and family members, and difficulty in accessing other services.^( 11 )^ Barbera et al. also described that patient with cancer near end of life have many visits to emergency department in the final 6 months and the final 2 weeks of life and related to the scope of palliative care expertise, highlights for control symptoms such as pain, dyspnea, nausea and vomiting, constipation, malaise, and fatigue.^( 15 )^

Here, we also hypothesized that the protective barriers established for this population during first wave, such telemedicine, could have reduced the comfort given to the family/ caregiver, and quality of care, what may have reflected in adherence.^( 14 , 16 , 17 )^In this way, Rossi et al. focused on the impact of COVID-19 doctor- patient communication describing the importance of physician express validation, empathy, and support in daily routine.^( 18 )^

To evaluate if the quality of assistance to these group of patients was influenced by pandemic scenario, we also compared records between the period mentioned- March 2020 to July 2020 to period before COVID-19 pandemic. When we consider care delivered to patients with advanced disease before and during the pandemic, 33.1% and 17.2%, respectively, were referred to the ICU in the last hospitalization. The difference between ICU reference was not statistically significant. although the value declined to almost half of previous valor. These could be related with external factors associated to the influence of pandemic in day care routine and hospital environment.^( 14 , 16 , 17 , 19 )^

In our study we could also observe that 34.4% of patients had documented their end-of-life care preferences before admission to hospital, whereas 13.8% during the pandemic. Although not statistically significant, we can also raise the hypothesis that, from March 2020 to July 2020, some conversations about prognosis may have been less frequent. In usual oncology care, physician probably would spend more time talking about life expectance and treatment plan to the patient, family, and caregivers and, as we mentioned before, empathy and support was not the same during these periods which could affect communication, mainly in difficult conversations. Delivering a patient-centered practice includes to put the patient’s needs, values, and preferences in the plan of care, sharing decision making, and ensuring effective communication in a interdisciplinary action. Thus, the reduction in time spent with the patient and the family could influence quality of assistance.^( 14 , 16 - 19 )^

Still, when we talk about quality of care for patients at the end of life, it is important to add to this period the presence of the palliative care professional, who is an important player in conversation about prognosis and relief of symptoms.^( 19 - 21 )^ While there were no differences in the proportion of patients seen by the palliative care team, before and during the pandemic, when time between the first visit of palliative care and death are considered, the mean days of documentation on end-of-life care preferences was 53.9 in the group with palliative care support *versus* 20.7 the group with no such support (p=0.0223).

Although, these finds not influenced statically on assistance. When we analyze care during the last days of life, we did not find statistically significant differences in the use of chemotherapy, radiation therapy, nutrition support, referral to ICU, or proportion in documentation on the end-of-life care preferences. Additionally, we observed no statistically significant differences related to admission to ICU and palliative care team support in both periods.

However, in previous analyses conducted by our service, where 111 consecutive patients with advanced cancer referred for the first time to an inpatient palliative care consultation were included and we demonstrated less frequent use of chemotherapy (39% *versus* 25%; p<0.001), more patients had end-of-life care preferences (39% *versus* 25%; p<0.001) and were not willing to be intubated (32% *versus* 60%; p<0.001), submitted to intensive care (30% *versus* 55%; p<0.001), cardiopulmonary resuscitation (35% *versus* 62%; p<0.001), and artificial nutrition (22% *versus* 34%; p<0.001).^( 21 )^

Comparative analysis between populations from other groups in pandemic scenario is difficult, since number of publications describing the group chosen to this study are small. However, considering previous pandemic, our results differ from other groups in time to referral to palliative assistance and ICU. Hui et al. showed admissions to ICU ranged from 5% to 11% associated with earlier palliative care referral,^( 22 )^ and Kim et al. reported 37.7% of admissions in patients without palliative assistance.^( 23 )^ These differences may again be explained by cultural barriers to referral to palliative care, since the gains described are directly related to early referral.^( 18 - 20 )^

In a prospective randomized study, Temel et al. evaluated the early integration of patients with recent diagnosis of lung and gastrointestinal tract cancer, with no curative proposals, between 2011 and 2015, describing gain in quality of life in the course of the disease, and reduction of depressive symptoms, associated with improved communication and alignment of care preferences.^( 24 )^

Despite the benefits observed to early referral palliative team, only 59% of patients in our study were referred to palliative group. Our results are similar to study conducted by Collins et al., on a 10-year evaluation of patients with lung cancer and their referral to palliative care, totaling up 46,700 cases, showed 59% received palliative care for a mean of 27 days before death.^( 25 )^

To understand the barriers to integrating palliative care and oncology practice, Dhollander et al. set up discussion groups on the subject, comprising professionals from the field in Belgium, and noted that even in a population considered expert, it was difficult to understand palliative care was not only about end-of-life care.^( 26 )^However, integrated activity has gains in any period, as we observed in our population, who even restricted to the end of life.^( 27 , 28 )^

Based on our results, we observed there are probably some barriers related to referral to palliative care, such as talking about prognosis. We also could observe that when referral to palliative team prognosis conversation are more frequent, although this find did not influenced in other quality measures. This result must be reevaluated for further studies with more balanced conditions in health system and, if possible, with less biases related to protective measures for COVID-19 pandemic. In this study we retrospectively analyzed medical records of a rather heterogeneous oncologic population in terms of diagnoses, although they all belonged to the group of advanced-stage neoplasms, and were treated by different physicians, which may add more bias.

## CONCLUSION

Despite the decrease in oncology admissions, the advanced-stage cancer patients with no COVID-19 continued to seek hospital for end-of-life care. Despite limitations described, our study demonstrates that the culture of dying in the hospital and safety related to his assistance was not influenced by the pandemic scenario. The feeling of protection seemed to be more expressive than the fear of hospitalization. However, we could observe in our benchmarking analyses for palliative quality of care, that talks about prognosis occurred less often during the pandemic.
